# Food hypersensitivity by inhalation

**DOI:** 10.1186/1476-7961-7-4

**Published:** 2009-02-20

**Authors:** Daniel A Ramirez, Sami L Bahna

**Affiliations:** 1 Allergy & Immunology Section, Louisiana State University Health Science Center in Shreveport, 1501 Kings Highway, Shreveport, LA, USA

## Abstract

Though not widely recognized, food hypersensitivity by inhalation can cause major morbidity in affected individuals. The exposure is usually more obvious and often substantial in occupational environments but frequently occurs in non-occupational settings, such as homes, schools, restaurants, grocery stores, and commercial flights. The exposure can be trivial, as in mere smelling or being in the vicinity of the food. The clinical manifestations can vary from a benign respiratory or cutaneous reaction to a systemic one that can be life-threatening. In addition to strict avoidance, such highly-sensitive subjects should carry self-injectable epinephrine and wear MedicAlert^® ^identification. Asthma is a strong predisposing factor and should be well-controlled. It is of great significance that food inhalation can cause *de novo *sensitization.

## Review

### Introduction

While much focus has been given to food hypersensitivity reactions following oral ingestion, reactions by skin contact and inhalation have slowly gained interest. Food allergy by the non-ingestant route is probably under-recognized and under-reported. This review provides a summary of the relevant studies and selected case reports on food allergy by inhalation and suggestions for management.

## Exposure Settings

Persons who come into contact with food, in either occupational or non-occupational settings, can inhale food particles that may lead to adverse reactions in highly sensitive individuals, or may cause *de novo *sensitization. Although the degree of exposure to food allergens is a major factor, concomitant environmental exposures to non-specific irritants can enhance the reactivity of the respiratory tract [1]. Exposure settings that may lead to reactions are listed in Table 1 and foods that have been implicated are listed in Table 2. Given the unpredictability and variety of these exposures, their identification by patients and clinicians can be challenging.

**Table 1:** Settings for food inhalation

**Occupational**
Farms
Food industry (bakery, confectionary, seafood processing)
Restaurants
Grocery stores
Food handling or processing
**Non-occupational**
Home
School
Airliners
Restaurants
Other

**Table 2:** Reported inhaled food allergens

**More commonly reported**
Wheat flour
Seafood (crustaceans more than bony fish)
Soy
Peanut
Hen's egg
Milk
**Less commonly reported**
Asparagus
Carrots
Tea leaves
Bell peppers
Garlic
Green beans
Seeds
White potato
Tomato
Rice
Carmine dye

Inhalation of food allergen depends on its airborne presence. This can occur, for instance, from the release of wet aerosols of snow crab allergens when they are "cracked" in seafood processing plants [2]. Processing of a food, such as boiling, steaming, or frying, can also release significant quantities of particulates into the air. This aerosolization has also been identified as a potential high risk factor for sensitization by inhalation [3]. In many patients, the reaction is dose-dependent [4].

## Manifestations of food reactions by inhalation

In the majority of patients, food particle inhalation induces respiratory symptoms that can be nasal (rhinorrhea, sneezing, nasal congestion), ocular (tearing, redness, irritation), or lower respiratory (cough, wheeze). In addition, skin manifestations and even, although much more rarely, anaphylaxis can occur.

### Rhinitis and/or conjunctivitis

Rhinoconjunctivitis is a common complaint associated with allergy to food by inhalation. Rhinitis and/or conjunctivitis may be the only manifestation, but they often precede more severe symptoms, as observed in the context of occupational asthma. As indicated by Malo et al [5], upper respiratory symptoms can often be regarded as early warning signs for future development of occupational asthma. Rossi et al [6] reported a 54-year-old man who worked as a kneader at a delicatessen factory. From age 25–40, his duties consisted of pouring meat into a grinder and sprinkling spices and sodium caseinate powder by hand onto the meat. By age 30, he began to develop oral symptoms of burning and itching following ingestion of dairy products. Two years later, he began to complain of nasal itching and stuffiness while at work. His symptoms resolved while he was away from work. Over the next ten years, his symptoms progressed necessitating his relocation to another department for cutting and grinding meat. He worked there for eight years without any symptoms until a kneading machine was installed near his work area. This precipitated acute onset of ocular itch, wheeze, and dyspnea. Skin prick testing was negative to common aeroallergens, but positive to both lactoglobulin and casein. Specific IgE was positive to lactoalbumin and casein. Bronchial challenge testing with sodium caseinate produced an immediate asthmatic response.

In certain exposure situations, the reaction to a plant can be to its pollen rather than to the actual fruit or vegetable. In a report on 209 greenhouse employees whose rhinitis symptoms were associated with the flowering season of green bell pepper, skin prick testing was positive to the plant's pollen in 47% [7]. An 8-year-old girl who had a history of anaphylaxis from ingestion of white potato had wheezing while walking in a white potato field during the pollen season [8].

### Cutaneous

Cutaneous manifestations of food hypersensitivity mostly occur by ingestion or skin contact. They can occasionally occur through inhalation. In a study of 197 children allergic to fish by ingestion, 21 showed symptoms by inhalation. The symptoms were respiratory (mostly wheezing) in 9, cutaneous (mostly urticaria) in 9, and combined in 3. The exposures occurred while fish was being fried in 14, boiled in 6, and served to others nearby in 5 [9].

### Asthma

Asthma is one of the most reported reactions to food by inhalation. In 1700, Bernardino Ramazzini published his *Treatise on Occupational Diseases*, where he described detailed observations of adverse reactions to inhaled foods in workers of various industries [10].

Continued increase in production and consumption of seafood worldwide has led to more frequent reporting of adverse reactions in both occupational and non-occupational settings. Reported prevalence of occupational asthma to seafood, for instance, ranged from 7% to 36% [3]. Seafood allergens, mostly heat stable high molecular weight proteins, can be aerosolized during trapping, processing, and/or cooking [11]. They are also resistant to these processes as well as to digestive enzymes. Approximately 30% of airborne particulates are small enough (≤ 5 μm) to reach the distal airways [3]. Pascual et al [12] demonstrated that steam emissions from boiling salmon contained allergenic proteins shared with raw and boiled salmon meat. Occupational asthma associated with inhalation of crustaceans is more prevalent than to bony fish and molluscs. While there are inconsistent cross-reactivities among bony fish species, there is a high degree of IgE cross-reactivity among crustaceans (shrimp, crab, lobster, and crawfish) [3].

Other foods have been implicated in causing IgE-mediated occupational asthma. Asparagus (*Asparagus officinalis*), a member of the Liliaceae family, is one such example. One report described a 28-year-old man who complained of rhinoconjunctivitis and asthma while harvesting raw asparagus, but was able to eat cooked asparagus without any symptoms [13]. His skin test to asparagus was positive, and bronchial challenge with raw asparagus extract produced an immediate asthmatic response. Two control individuals with seasonal allergic asthma who were not exposed to asparagus did not react to a similar challenge. Garlic, also a member of the Liliaceae family, is known to be a potent skin contact allergen, and has been implicated in IgE-mediated reactions by inhalation. Añibarro et al [14] studied 12 patients with rhinoconjunctivitis and/or asthma who were exposed to garlic dust as harvesters, in storage facilities, or in spice factories. Over half of these patients had immediate response (rhinitis, asthma, or both) to bronchial challenge with garlic.

Asthma to food inhalation has been reported in non-occupational settings. Through a telephone survey, Sicherer et al [15] described patients who had adverse reactions to peanuts aboard commercial flights. In 33% of patients, the reaction was by inhalation. In the majority of patients, the symptoms were rhinorrhea and wheezing. No anaphylaxis was reported. Half of the patients had a history of asthma. Through a more recent telephone survey, Comstock et al [16] reported on reactions to peanuts and tree nuts in 41 patients (68% had history of asthma) during flights, 58% of which were by inhalation. The reactions to peanuts were about four times more than tree nuts (73% vs. 18%), probably because of more commonplace peanut distribution. Many of the reactions by inhalation were classified as severe, though none were anaphylactic. Although 32 of these patients had been previously instructed to carry self-injectable epinephrine, only twelve (38%) had it with them on the flight. In many patients, the reactions by inhalation were apparently more severe than by ingestion; a similar finding was reported in fish [17]. There is evidence that food protein allergenicity is reduced by gastric digestion[18]

Some concern has been raised about food allergic reactions on commercial flights and in schools. While there has been some debate on possible legislation banning peanuts aboard commercial flights by the Federal Aviation Administration, no mandatory policy exists at present. Most carriers have elected not to serve peanut snacks altogether. Our personal opinion is that it would be difficult and unnecessary to enforce universal banning of a specific food on flights or in schools. The number of affected individuals is so small and preventive measures can be applied, as we propose in Table 3.

**Table 3:** Recommended management of subjects with hypersensitivity to food inhalation

**Measures by the subject**
Strict avoidance of the offending foods
Prepare own food
Take long-acting antihistamine before potential accidental exposure
Carry self-injectable epinephrine (2 doses), fast-acting antihistamine, and albuterol inhaler
Asthma should be well-controlled
Wear MedicAlert^® ^identification
**Measures by airlines or schools**
Provide a substitute snack for affected persons
Carry emergency treatment measures
Educate their personnel on food allergy

Green bean hypersensitivity by inhalation has been reported in a homemaker [19]. She experienced asthma and rhinitis while boiling raw green beans, but had no symptoms upon ingestion of cooked green beans. Specific IgE testing was positive to raw green bean extract. Though skin prick testing was negative to boiled green beans, and only weakly positive to heated green bean extracts, bronchial challenge testing showed immediate reaction to both heated and unheated extracts. A five-year-old boy was so exquisitely sensitive to peanut odor that it was strictly banned from his classroom. However, he developed acute wheezing upon entering the classroom of a substitute teacher who had just eaten peanuts [20].

### Systemic anaphylaxis

It is worth noting that systemic anaphylaxis to food allergens is very rare, yet striking examples have been reported. One report described an 11-year-old boy who had anaphylaxis while his mother was cooking rice. He was able to consume rice without any symptoms, but bronchial challenge with rice induced anaphylaxis [21]. Another report described anaphylaxis in an 8-year-old girl while white potatoes were being boiled at home [8]. IgE-mediated allergy to potato was evidenced in her by positive skin prick, specific IgE, basophil histamine release, and passive transfer testing. A 6-year-old boy with multiple food allergies had anaphylaxis from being merely in the vicinity of fried fish and urticaria while in the vicinity of uncooked fish or egg [22]. Skin prick testing and specific IgE were strongly positive to egg, fish, shrimp, and crab. Fatal anaphylaxis occurred in a woman after walking through a milk storage room in a barn [23]. Her serum specific IgE was known to be positive to casein. A 33-year-old male on a milk-free diet due to milk allergy in childhood complained of shortness of breath with loss of consciousness while he was milking sheep. He had positive skin prick and specific IgE tests to casein, lactoglobulin, and whole milk. He later discovered that wearing a gas mask while milking prevented further attacks [24].

Anaphylaxis has been reported to inhaled medications containing trace amounts of food proteins. Some formulations of inhaled ipratropium bromide include soya lecithin and may cause reactions in patients allergic to soybean and peanut. Anaphylaxis occurred in a woman with severe peanut allergy following nebulization with ipratropium bromide solution [25]. In another unexpected case [26], an 8-year-old boy with severe milk allergy and persistent asthma had repeated anaphylaxis after his intake of fluticasone/salmeterol dry powder inhaler. He had tolerated this medication previously. The new inhaler was found to contain trace amounts of lactose. Lactose is derived from bovine milk and is commonly used in pharmaceuticals. Since some inhaled medications may contain food proteins, it would be prudent to advise highly allergic subjects to carefully read labels, even of medications used to treat asthma.

Conditions such as panic attacks and anxiety could be mistaken for anaphylaxis, hence the importance of recording the heart rate, blood pressure, and the presence of associated signs, e.g., urticaria, angioedema, or wheezing. When a psychological component is suspected, blind placebo-controlled testing would be most helpful.

### Unique reactions

Some foods can produce reactions by inhalation only, yet be well-tolerated by ingestion. *Baker's asthma *is a peculiar type of occupational asthma, affecting up to 9% of bakers, particularly in Europe [27]. It is the leading cause of occupational asthma in France [28], and the second highest in the Great Britain [29]. In addition to bakers, any worker exposed to bakery allergens, such as confectioners, flour millers, and food processors, can develop the disease [30]. Bakery allergens typically consist of wheat flour and fungal enzymes. In addition, rye flour has gained increased attention as a causative inhaled allergen in baker's asthma, a phenomenon attributed to its increased cultivation and use [31]. In fact, 60–70% of bakery workers with rhinoconjunctivitis and asthma were found to have elevated specific IgE levels to wheat, rye, or both extracts [32]. Interestingly, patients who have baker's asthma usually ingest wheat products with impunity [33].

Hypersensitivity pneumonitis, primarily mediated by type II and type IV hypersensitivity reactions, has been described in a 45-year-old woman [34]. She was a veterinary diet researcher who had chronic respiratory symptoms and recurrent hospitalizations for episodes of fever, dyspnea, chills, hypoxemia, leukocytosis, and pulmonary infiltrates. Her work involved using soybean. Her immunologic, serologic, and microbiologic evaluations were normal. A bronchoalveolar lavage showed increased macrophages and polymorphonucleocytes. A lung biopsy showed lymphocytic interstitial infiltrate and alveolar fibrinous hemorrhagic exudate. Specific IgE, specific IgG, and precipitins were negative to bird droppings, fungi, animal dander, and soybean free animal fodders. Delayed hypersensitivity skin testing and serum precipitating antibodies were positive only to soybean-containing animal fodder. After three months away from work, she was asymptomatic. A bronchial challenge with one nanogram of soybean led to a precipitous episode of pneumonitis.

## Mechanisms of reactions by inhalation

Food allergic reactions by inhalation occur in subjects who are already sensitized to the food, generally by ingestion. The reverse, however, can occur, i.e., *de novo *sensitization through inhalation. Reactions by first ingestion have been reported in patients whose previous exposure to the food was by inhalation. A double-blind, placebo-controlled study demonstrated positive oral challenge results to lupine seed flour in workers at an agricultural research center who had been exposed to lupine seed flour by inhalation [35]. Another report described six bakery workers who suffered from rhinoconjunctivitis and four from asthma on exposure to airborne egg proteins [36]. Of the four who complained of asthma, three later reported symptoms following ingestion of egg-containing food products. Sensitivity to egg protein was positive in four subjects by percutaneous testing, and in all six by specific IgE testing. A final example comes from a patient who was exposed to dust of seeds from feeding birds, who later developed oral allergy symptoms upon eating sunflower seeds [37]. Severe reactions by ingestion following sensitization by inhalation can occur. One instance was a 60 year-old woman who had systemic anaphylaxis after eating a cereal containing psyllium. Her only previous known exposure to psyllium had been dispensing psyllium-containing laxative powder as a nurse four years earlier. In addition to a positive skin prick test to psyllium, in vitro basophil histamine release was positive to psyllium and was much reduced after desensitization of the cells with anti-IgE [38]. Another woman with a similar story had severe laryngeal edema and fatal anaphylaxis following ingestion of psyllium-containing product for the first time [39]. She had previously worked as a nurse assistant preparing psyllium bulk laxatives for years. Specific IgE was strongly positive only to psyllium, while the remaining twenty one ingredients of the ingested food product were negative. Her serum tryptase level was elevated, indicating mast cell degranulation.

Provoked reactions by inhalation to foods can occur without any known history of ingestion of the offending food. One case series in France revealed eight children who had asthmatic reactions following inhalation of peanut [40]. All demonstrated evidence of sensitization to peanut, though none had a history of overt ingestion of peanut. Another report described a boy who at 5 years of age developed wheezing upon exposure to shrimp that was being cooked, even though he had no known record of ever eating seafood. At age 7, his total IgE was highly elevated, and specific IgE was strongly positive to egg yolk, crab, and shrimp [20].

## Conclusion

Patients who are allergic to food by ingestion may react to the same food by inhalation. The inhaled quantity can be substantial, particularly in certain occupational settings, or very trivial as by mere smelling. Asthma is a strong predisposing factor. The manifestations may be limited to the respiratory tract or involve other systems, including, although rarely, systemic anaphylaxis. Such patients should be advised against any exposure to the offending food, carry self-injectable epinephrine, and wear MedicAlert^® ^identification. Concomitant asthma should be well controlled. Whereas food inhalation is commonly recognized as symptom-provoking in patients who have already developed food allergy, it can cause *de novo *sensitization, particularly from chronic exposure in occupational settings.

## Competing interests

The authors declare that they have no competing interests.

## Authors' contributions

DAR collected and reviewed the literature and shared in preparing the manuscript. SLB selected the topic and shared in preparing the manuscript.
